# Highly efficient and expedited hepatic differentiation from human pluripotent stem cells by pure small-molecule cocktails

**DOI:** 10.1186/s13287-018-0794-4

**Published:** 2018-03-09

**Authors:** Cong Du, Yuan Feng, Dongbo Qiu, Yan Xu, Mao Pang, Nan Cai, Andy Peng Xiang, Qi Zhang

**Affiliations:** 1 Guangdong Provincial Key Laboratory of Liver Disease Research, The Third Affiliated Hospital of Sun Yat-sen University, Guangzhou, 510630 People’s Republic of China; 2 Cell-gene Therapy Translational Medicine Research Center, The Third Affiliated Hospital of Sun Yat-sen University, Guangzhou, 510630 People’s Republic of China; 3 Center for Stem Cell Biology and Tissue Engineering, Key Laboratory for Stem Cells and Tissue Engineering, Ministry of Education, Sun Yat-Sen University, Guangzhou, 510080 People’s Republic of China; 4 Department of Spine Surgery, The Third Affiliated Hospital of Sun Yat-sen University, Guangzhou, 510630 People’s Republic of China; 5 Biotherapy Center, The Third Affiliated Hospital of Sun Yat-sen University, Guangzhou, 510630 People’s Republic of China; 6 Biotherapy Center & Cell-gene Therapy Translational Medicine Research Center, Guangdong Provincial Key Laboratory of Liver Disease Research, The Third Affiliated Hospital of Sun Yat-sen University, Guangzhou, 510630 China

**Keywords:** Hepatocyte differentiation, Human pluripotent stem cells, Small molecules

## Abstract

**Background:**

The advent of human-induced pluripotent stem cells holds great promise for producing ample individualized hepatocytes. Although previous efforts have succeeded in generating hepatocytes from human pluripotent stem cells in vitro by viral-based expression of transcription factors and/or addition of growth factors during the differentiation process, the safety issue of viral transduction and high cost of cytokines would hinder the downstream applications. Recently, the use of small molecules has emerged as a powerful tool to induce cell fate transition for their superior stability, safety, cell permeability, and cost-effectiveness.

**Methods:**

In the present study, we established a novel efficient hepatocyte differentiation strategy of human pluripotent stem cells with pure small-molecule cocktails. This method induced hepatocyte differentiation in a stepwise manner, including definitive endoderm differentiation, hepatic specification, and hepatocyte maturation within only 13 days.

**Results:**

The differentiated hepatic-like cells were morphologically similar to hepatocytes derived from growth factor-based methods and primary hepatocytes. These cells not only expressed specific hepatic markers at the transcriptional and protein levels, but also possessed main liver functions such as albumin production, glycogen storage, cytochrome P450 activity, and indocyanine green uptake and release.

**Conclusions:**

Highly efficient and expedited hepatic differentiation from human pluripotent stem cells could be achieved by our present novel, pure, small-molecule cocktails strategy, which provides a cost-effective platform for in vitro studies of the molecular mechanisms of human liver development and holds significant potential for future clinical applications.

**Electronic supplementary material:**

The online version of this article (10.1186/s13287-018-0794-4) contains supplementary material, which is available to authorized users.

## Background

Orthotopic liver transplantation (OLT) is the only effective treatment for end-stage liver diseases, but there is currently a severe shortage of liver grafts available for transplantation [1]. Functional hepatocytes not only have therapeutic value for regenerative medicine and pharmacology research but also may be an attractive alternative to OLT [2, 3]. Human primary hepatocytes are a ideal cellular resources for achieving these aims. However, the utility of primary hepatocytes in either pharmaceutical or clinical applications is hindered by their limited sources, lack of proliferative ability and rapid decline of functions over extended culture periods in vitro [4, 5], which has also been verified by our own experiments. Hepatocyte-like cells (HLCs) differentiated from human embryonic stem cells (hESCs) or induced pluripotent stem cells (hiPSCs) bring new hopes to overcome these difficulties [6–8]. Thus, substantial efforts have been devoted to generating functional hepatocytes from human pluripotent stem cells (hPSCs) [9–15].

The earliest strategy for generating hepatocytes from PSCs involved the formation of embryoid bodies (EBs), a highly inefficient and heterogeneous process [16]. Later, different groups have achieved remarkable improvements in differentiation efficiency and cellular functions either by sequential transduction of hepatic-specific transcription factors (such as FOXA2, FOXA3, GATA4, hepatocyte nuclear factor-4α (HNF4α), and so on) into PSCs [17, 18], or stepwise titration of cytokines and growth factors mimicking liver developmental signaling pathways in vivo [like Wnt3a, activin A, fibroblast growth factor 4 (FGF4), bone morphogenetic protein 4 (BMP4), hepatocyte growth factor (HGF), and oncostatin M (OSM)] [14, 19–23]. Cells generated by most protocols showed encouraging results of functions in vitro and even the ability to survive and repopulate the liver function after being transplanted into animal models [9, 10, 14, 15, 19].

Although more advances on hepatic differentiation were reported, there still remain a lot of problems and discrepancies [24, 25]. For example, the maturation of most differentiated cells so far needs to be improved compared to their in vivo counterparts. Additionally, it usually took 15 days to 1 month to get functional hepatocyte-like cells using six or more cytokines [14, 22, 23, 26]. The long duration and big consumption of cytokines can hardly meet the requirements for large-scale production of cells in clinical and pharmacological application. Small molecular chemical compounds may offer a promising alternative to overcome these issues since they are effective to interfere with signals involved in early development and showed potential to improve the synchronization and efficiency of PSC differentiation [27–29]. With the progressive understanding of signals controlling liver differentiation and development of more target-specific small molecules, it becomes feasible to manipulate cell fate in vitro with pure chemical compounds [30].

Wnt signaling activation is indispensable for the definitive endoderm (DE) formation, the first step of hepatocyte differentiation from hPSCs [31, 32]. Our preliminary data showed that activation of Wnt pathway by glycogen synthase kinase (GSK)-3β inhibitor (such as CHIR99021) is beneficial for DE differentiation, which is consistent with others’ reports [33, 34]. Dimethyl sulfoxide (DMSO) alone has been found to be able to induce DE differentiation toward hepatic progenitors [34]. However, in preliminary experiments, we found that 1% DMSO caused obvious toxicity to cells and was not sufficient to induce hepatic differentiation. In this study, we designed a novel cost-effective strategy to direct the hepatic differentiation from hPSCs (including hESCs or hiPSCs) using only commercially available small molecules. The hepatocytes generated by our pure small-molecule-driven approach expressed high levels of hepatocyte-specific markers and displayed the important biological functions of liver. Our present work may provide a novel strategy generating hPSC-differentiated hepatocytes efficiently for drug screening, disease modeling, and cell therapy.

## Methods

### Culture of human pluripotent stem cells

Human pluripotent stem cells (hESCs-H1, H7, and hiPSCs) were identified and characterized as previously reported [35–37]. They were maintained as colonies on tissue culture plates pre-coated with Matrigel (Corning Life Sciences, Corning, NY, USA) in mTeSR™1 medium (Stem Cell Technologies, Vancouver, BC, Canada), which is a chemically defined and feeder-free culture medium being widely used, at 37 °C in a 5% CO_2_ incubator. Human PSCs were passaged every 4–5 days by incubation with the enzyme-free passaging reagent ReLeSR™ (Stem Cell Technologies) for 5 min at 37 °C according to the manufacturer’s instructions. The colonies were resuspended in mTeSR™1 medium and replated at split ratios ranging from 1:3 to 1:9 as appropriate.

### Hepatocyte differentiation in vitro

When human PSCs reached a confluency level of approximately 80%, they were passaged with StemPro® Accutase® Cell Dissociation Reagent (Thermo Fisher Scientific, Waltham, MA, USA) and resuspended as single cells in mTeSR™1 medium. The cells were seeded in six-well plates pre-coated with Matrigel diluted in DMEM/F12 (Thermo Fisher Scientific) for at least 1 h at 37 °C in a CO_2_ incubator. For initial differentiation, the expansion medium was changed to 0.5% DMSO (Sigma-Aldrich, St. Louis, MO, USA) in mTeSR™1 medium. After 24 h, the pretreatment medium was switched to RPMI 1640 (Thermo Fisher Scientific) with B27 Supplement Minus Insulin (Thermo Fisher Scientific), along with 3 μM CHIR99021 (Selleck, Houston, TX, USA). Following 24-h treatment, CHIR99021 was withdrawn and the cells were treated with RPMI 1640/B27 basal medium alone for another 24 h. The differentiated cells were cultured in Advanced F12 basal medium (Thermo Fisher Scientific) with A83–01 (0.5 μM; Selleck), sodium butyrate (250 nM; Sigma-Aldrich) and dimethyl sulfoxide (0.5% of total volume) for 5 days. The culture medium was changed daily. For hepatocyte generation in the last stage, the differentiation medium was switched to Advanced F12 basal medium supplied with five commercial small molecules, containing FH1 (15 μM), FPH1 (15 μM), A83–01 (0.5 μM), dexamethasone (100 nM) and hydrocortisone (10 μM). All of the above-mentioned small molecules were purchased from Selleck. Advanced F12 basal medium was composed of Advanced DMEM/F-12 medium (95% of total volume), B-27 Serum-Free Supplement (1% of total volume), KnockOut™ Serum Replacement (1% of total volume), GlutaMAX™ Supplement (1% of total volume) and MEM Non-Essential Amino Acids Solution (1% of total volume), all of the components were purchased from Thermo Fisher Scientific. The cells were collected and analyzed at each differentiation stage. The small molecules used in our protocol are listed in the Additional file 1: Table S1. For the growth factor-induced hepatic differentiation protocol, we used previously described protocols [14, 21, 23]. Briefly, activin A (100 ng/ml) was used to induce the definitive endoderm from human PSCs. Bone morphogenetic protein 4 (BMP4) (10 ng/ml) and fibroblast growth factor 4 (FGF4) (10 ng/ml) were used to induce hepatic specification from the definitive endoderm. All growth factors were purchased from Peprotech Co. (Rocky Hill, NJ, USA).

### Isolation of adult human primary hepatocytes

Adult human primary hepatocytes used in this study were isolated from donation after cardiac death (DCD) during liver transplantations under the approval of the Medical Ethics Committee of the Third Affiliated Hospital of SYSU. The written informed consent was obtained in accordance with the institutional review board guidelines before liver transplantation surgery. Human primary hepatocytes were isolated following a two-step perfusion protocol. In brief, the liver sample was firstly perfused with warm D-Hanks buffer for 15 to 30 min and then with collagenase H (*Clostridium histolyticum*) (0.1 mg/ml, Sigma-Aldrich) in warm Hank’s buffer for another 15 to 30 min. The liver sample was transferred to a 100-mm cell-culture dish containing 10 mL of ice-cold high-glucose DMEM medium and the cells dispersed further through a large-bore pipette. The cell suspension was filtered through 70-μm nylon cell-strainer and centrifuged at 50 g for 5 min at 4 °C. After the second wash and centrifuge, the supernatant was carefully removed and the cell pellet resuspended gently with high-glucose DMEM medium which contained 10% of fetal bovine serum (FBS). The hepatocytes were seeded on a 6-well plate which was coated with Collagen I (5 μg/cm^2^, Thermo Fisher Scientific) ahead of time. The cells were incubated at 37 °C with 5% CO_2_ for 4 h and cell attachment was checked under the microscope. Most of the cells were viable and attached to the bottom of the tissue-culture plate. For the following experiments, human primary hepatocytes were used as positive control.

### RNA purification and real-time-PCR

An RNA isolation and purification Kit (Omega Bio-tek, Norcross, GA, USA) was used to extract RNA from cultured cells. The amount and quality of the RNA were determined using a BIOMATE 3S UV-visible spectrophotometer (Thermo Fisher Scientific). cDNA synthesis was performed with 1 μg of RNA using PrimeScript reverse transcriptase (Takara, Tokyo, Japan) and reverse-transcribed using a PCR Instrumentation C1000 Touch™ Thermal Cycler (Bio-Rad Laboratories, Hercules, CA, USA) according to the manufacturers’ protocol. The cDNA was then amplified by fluorescent quantitative PCR (qPCR). Q RT-PCR analysis was performed on an ABI Prism 7500 Sequence Detection System using the SYBR Green PCR Master Mix (Applied Biosystems, Carlsbad, CA, USA). Additional file 1: Table S2 shows the primer pairs used in our study. The gene expression of pluripotent markers (OCT4, NANOG), DE markers (sex determining region Y (SRY)-box 17 (SOX17), FOXA2), mesoderm markers (HAND1, BRA), ectoderm markers (GAP43, ZIC1), hepatic progenitor markers [alpha-fetoprotein (AFP), HNF4α, cytokeratin 18 (CK18), cytokeratin 19 (CK19)], and hepatocyte markers [albumin (ALB), alpha-1 antitrypsin (A1AT), apolipoprotein A2 (APOA2), ASGR1, CYP1A2, CYP2B6, CYP3A4, and so on] were measured. All data were presented as the mean of at least three independent experiments. The error bars represent the standard deviation (SD). GAPDH expression was used as an internal control.

### Immunofluorescence microscopy

Cells at each differentiation stage were fixed with iced methanol or 4% paraformaldehyde for 15 min at room temperature and blocked with phosphate-buffered saline (PBS) containing 0.1% Triton X-100 and 3% bovine serum albumin (BSA) at room temperature for 1 h. Cells were then incubated with the appropriate primary antibodies at 4 °C overnight. On the second day, after three washes for at least 5 min with PBS, Alexa Fluor-conjugated secondary antibody diluted 1:1000 was added and incubated at room temperature for 1 h. Hoechst 33342 (Thermo Fisher Scientific) diluted in 1:5000 was used to stain the cell nuclei. Between each step, cells or sections were washed with fresh PBS. Image acquisition and processing were carried out using a fluorescence microscope (Zeiss LSM 800 and Axio Observer, Carl Zeiss Microscopy, Jena, Germany). The alpha fetoprotein (AFP)- and hepatocyte nuclear factor-4α (HNF4α)-positive cells and whole cells counterstained by Hoechst were counted by Image-Pro Plus software (Media Cybernetics, Rockville, MD, USA). Detailed information of all antibodies used in the immunofluorescence staining experiments was listed in Additional file 1: Table S3.

### Western blot analysis

Cells were lysed in ice-cold RIPA cell buffer (Teknova, Hollister, CA, USA) supplemented with a protease-inhibitors cocktail (Thermo Fisher Scientific). After centrifuging at 12,000 rpm for 10 min at 4 °C, the supernatant was collected as the total cell lysate. Equal amounts of protein were resolved by 10% SDS-PAGE gel and electro-transferred to nitrocellulose membranes (EMD Millipore, Burlington, MA, USA). The membrane was blocked with 5% nonfat milk for 1 h at room temperature, incubated overnight at 4 °C with the relevant primary antibodies, and then incubated with horseradish peroxidase-conjugated secondary antibodies for 1 h at room temperature. Between each step, the nitrocellulose membranes were washed with fresh Tris-Buffered Saline Tween-20 (TBST). The immunoreactive bands were detected with an enhanced chemiluminescence kit (Sigma-Aldrich). Detailed information of all antibodies used in the western blot experiments were listed in Additional file 1: Table S3.

### Flow cytometry

Cells were dissociated with Accutase (Thermo Fisher Scientific) and stained with the appropriate antibodies according to the manufacturer’s instructions. Briefly, for the detection of nuclear antigens, the cells were first fixed/permeabilized and subsequently incubated with APC- or PE-conjugated antibodies for 45 min in the dark at 4 °C. Each analysis was performed on at least three separate cell preparations. Detailed information of antibodies used in the flow cytometry experiments were listed in Additional file 1: Table S3. The stained cells were recovered using FACSCanto (BD Pharmingen, San Diego, CA, USA). Data were recorded using the BD FACS Diva Software program (BD Pharmingen) and analyzed using the FlowJo 7.6.1 program (Tree Star, Ashland, OR, USA).

### Periodic acid-Schiff staining for glycogen

Periodic acid-Schiff (PAS) is a staining method that is primarily used to identify glycogen storage in cells. Cells were fixed in 4% paraformaldehyde and stained using a PAS staining system (Sigma-Aldrich) at room temperature. Briefly, fixed cells were oxidized with 1% periodic acid solution, then incubated in Schiff’s reagent. After being rinsed with PBS, cells were stained with Mayer’s hematoxylin. Between each step, the cells were washed with fresh PBS.

### Cellular uptake and release of indocyanine green

Indocyanine green (ICG) is a cyanine dye that can be taken in and released exclusively by mature hepatocytes and is used clinically to test hepatic function. ICG (Sigma-Aldrich) was dissolved in DMSO to prepare the stock solution with 5 mg/ml and was freshly diluted in culture medium to 1 mg/ml as the working solution. Cells were incubated in diluted ICG for 30 min at 37 °C. Cells were then rinsed three times with PBS and cellular uptake of ICG was examined by light microscopy. Cells were then returned to fresh culture medium, incubated for 6 h, and then examined with the phase contrast microscopy.

### Albumin secretion ELISA assay

At the endpoint of the differentiation process, the supernatant of cultured cells was collected. Albumin secretion in the supernatant was measured with a human albumin enzyme-linked immunosorbent assay (ELISA) quantitation kit (Bethyl, Montgomery, TX, USA) according to the manufacturer’s instructions. Cells were trypsinized and counted with Cellometer Auto T4 Bright Field Cell Counter (Nexcelom Bioscience, Lawrence, MA, USA). The albumin secretion was normalized to total cell numbers.

### Alpha fetoprotein secretion assay

The human alpha fetoprotein (AFP) content in the supernatant was determined with electrochemiluminescence immunoassay (Elecsys 2010, Roche Diagnostics, Basel, Switzerland) according to the manufacture’s protocol. Cells were trypsinized and counted with Cellometer Auto T4 Bright Field Cell Counter (Nexcelom Bioscience). The AFP secretion was normalized to total cell numbers.

### Cytochrome P450 activity

CYP1A2 activity was measured using a CYP1A2-MROD Assays kit (Genmed Scientifics, Arlington, MA, USA). The assay utilizes a nonfluorescent CYP1A2 substrate that is converted into a highly fluorescent metabolite (resorufin) detected in the visible range (Ex/Em = 530/590 nm). For CYP1A2 induction, omeprazole (100 μM) was added to the differentiated human ES and iPS cells during the last 3 days and human primary hepatocytes for 72 h. The medium was refreshed every day. The cells were lysed with RIPA (Thermo Fisher Scientific) and then homogenized with an ultrasonic crusher (Sonifier 450D, Branson Ultrasonics, Danbury, CT, USA). The assay was performed according to the manufacturer’s instructions. The fluorescence was measured with Multi-Detection Microplate Reader Spark 10 M (Tecan Group, Zürich, Switzerland). Cytochrome activity was normalized to the total protein (mg) and presented as pmol/mg protein/min.

### Statistical analysis

All data were obtained from at least three independent experiments, presented as means ± SD and analyzed by using the statistical software SPSS17.0 (IBM Corp., Armonk, NY, USA). The Student’s *t* test was used to compare the differences between two groups. *P* < 0.05 was considered statistically significant (^*^p < 0.05).

## Results

### Glucogen synthase kinase 3β (GSK-3β) inhibition promote definitive endoderm differentiation from human PSCs

We aimed to develop a novel differentiation strategy based on pure small molecules to acquire hepatocytes from human PSCs. The differentiation process involves three stages, including definitive endoderm differentiation, hepatic specification, and hepatocyte maturation. Human iPSCs were established and used in most experiments in this study. Similar experiments were also performed with the hESC-H1 and H7 cell lines and consistent results were obtained.

**Fig. 1:**
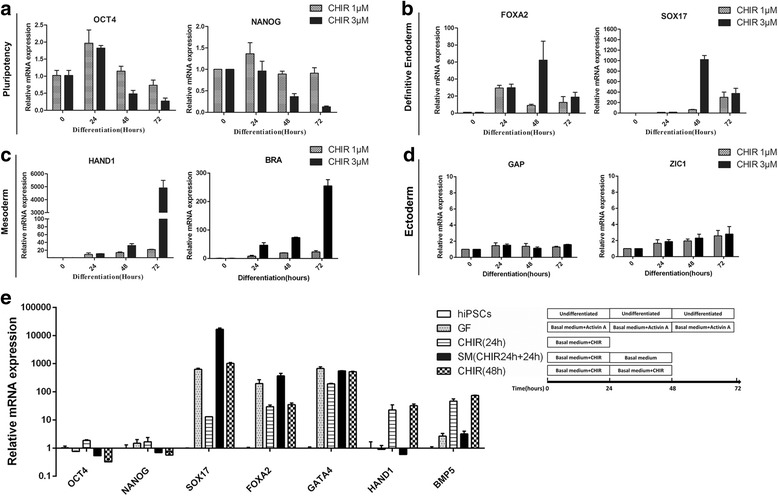
Optimization of concentration and duration of CHIR99021 treatment during DE induction. qRT-PCR for indicated genes using RNA lysates from human iPSCs treated with CHIR99021 at 1 μM or 3 μM for 24, 48, and 72 h during differentiation. Relevant expression of markers for pluripotency (**a**), DE (**b**), mesoderm (**c**) and ectoderm (**d**) were showed. **e** qRT-PCR for pluripotent markers (OCT4, NANOG), DE markers (SOX17, FOXA2), mesoderm markers (HAND1, BRA), and ectoderm markers (GAP43, ZIC1) using RNA lysates from human iPSCs exposed to CHIR99021 continuously or intermittent for 48 h. Growth factor-based method (activin A) was used for comparison

Based on the fact that Wnt/β-catenin signaling regulates sex-determining region Y (SRY)-box 17 (SOX17) expression and is essential for the formation of definitive endoderm [38], we set out to investigate whether CHIR99021 (CHIR), an inhibitor of GSK3β which can indirectly activate Wnt/β-catenin signaling, could promote definitive endoderm differentiation from hPSCs. Human iPSCs were treated with different concentrations of CHIR continuously for 72 h. Decreased expression of pluripotency transcription factors was observed in a dose-dependent manner (Fig. 1a). However, 9 μM or higher concentration of CHIR showed obvious toxicity and caused massive cell death (data not shown), while 1 μM could not induce differentiation efficiently (Fig. 1a). Thus, 3 μM was chosen as the optimal concentration in the subsequent experiments. In contrast to published protocols using RPMI 1640 and B-27 Supplement as the basal medium [34], we also changed the basal medium to RPMI 1640 and B-27 Supplement Minus Insulin to improve the definitive endoderm generation efficiency. After treatment with 3 μM CHIR, the mRNA levels of pluripotency markers were downregulated in a time-dependent manner (Fig. 1a). Interestingly, the gene expression of DE-specific transcription factors reached a peak after 48 h of treatment with CHIR and declined with further treatment (Fig. 1b). Furthermore, mesoderm- and ectoderm-related genes were upregulated in a time-dependent manner (Fig. 1c and d), consistent with previous reports that longer treatment with CHIR led to mesoderm derivation from PSCs [39].

**Fig. 2:**
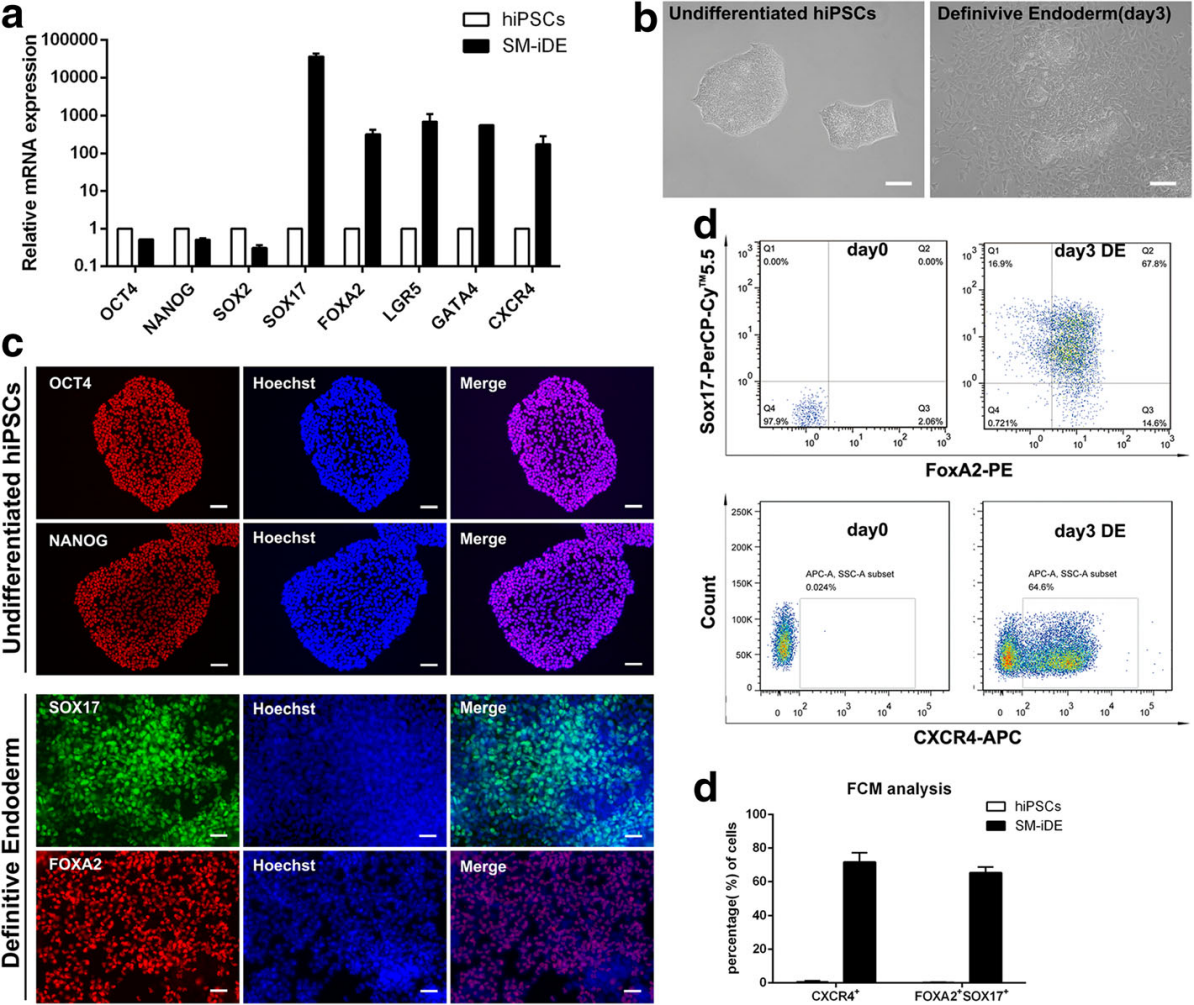
Small molecules efficiently induce definitive endoderm differentiation from hPSCs. **a** qRT-PCR for pluripotent markers and DE markers using RNA lysates from human iPSCs treated with DMSO at day 1, CHIR99021 (3 μM) at day 2, and then basal medium without CHIR99021 at day 3. **b** Phase contrast photos (×200) showing morphological changes during stage I of differentiation. Scale bars = 100 μm. **c** Immunofluorescence of pluripotency and DE-specific markers at the end of differentiation stage I. Scale bars = 100 μm. **d**, **e** Percentage of FOXA2/SOX17- and CXCR4-positive cells at day 0 and day 3 of DE differentiation analyzed by flow cytometry. **e** Histogram of the FOXA2/SOX17- and CXCR4-positive cells at day 0 and day 3 of DE differentiation analyzed by flow cytometry. Undifferentiated human iPSCs were used as control. All data were presented as the mean of at least three independent experiments. The error bars represent the SD

Continuous treatment with CHIR for 48 h had a negative influence on the ultimate hepatic differentiation efficiency, since unwanted mesoderm related markers heart and neural crest derivatives expressed 1 (HAND1) and bone morphogenetic protein 5 (BMP5) upregulated (Fig. 1c). Therefore, the CHIR treatment was terminated after 24 h, followed by treatment with basal medium for the subsequent 24 h. After these treatments, the pluripotency-related transcription factors were downregulated and the DE-specific markers were upregulated, while mesoderm-related markers were much lower compared to CHIR treatment for continuous 48 h, suggesting that human PSCs were inclined to differentiate into DE cells after 24 h treatment with CHIR (Fig. 1e). In order to improve the DE differentiation efficiency, we and other researchers found that dimethyl sulfoxide (DMSO) was beneficial for stem cell differentiation [22]. The optimal concentration of DMSO is very important as it is toxic to cultured cells at high concentrations. Concentrations of DMSO from 0.25% to 1% were tested and 0.5% was found to be the optimal concentration (data not shown). Based on these data, the first stage of the differentiation protocol was performed by pre-treating human PSCs with 0.5% DMSO for the first day and then 24 h with 3 μM CHIR followed by treatment with basal medium for another 24 h. At the 72-h time point (stage I endpoint), there was dramatically elevated expression of DE-specific transcription factors (Fig. 2a), at similar levels to those observed during growth factor-induced DE differentiation (Fig. 1e). These changes in gene expression were accompanied by morphologic changes from a dense cluster to a petal-like morphology (Fig. 2b) and elevated expression of FOXA2 and SOX17 at protein level (Fig. 2c and d). The efficiency of the DE formation using small molecules was up to 80% characterized by the expression of DE-specific markers CXCR4, FOXA2, and SOX17 (Fig. 2d, and e).

Collectively, the results demonstrated that the small-molecule cocktail could suppress pluripotency and drastically increase the expression of DE-related markers in hPSCs. Hence, for the subsequent small-molecule-based method to generate hepatic progenitor cells and hepatocytes, a combination of DMSO and CHIR was used for the first stage of DE induction.

### Hepatic-specific differentiation was achieved through transforming growth factor-β (TGF-β) inhibitor along with sodium butyrate and DMSO

Previously, we and others have found that sodium butyrate (SB), a known histone deacetylase inhibitor, could promote hepatic specification [22, 40]. In vivo, the liver and pancreas are derived from a common posterior foregut that develops from DE. Numerous publications have reported that TGF-β pathway activation was not beneficial for hepatic differentiation but was conducive to pancreatic differentiation. Based on the fact that each of the signaling pathways reciprocally represses the formation of the other lineage, we proposed that inhibiting the TGF-β pathway to repress pancreas formation could specifically lead to hepatic lineage generation with high efficiency. Therefore, the second stage of the small-molecule treatment involved treating the hPSC-derived DE cells with A83–01, an inhibitor of the TGF-β pathway, in combination with SB and DMSO.

**Fig. 3:**
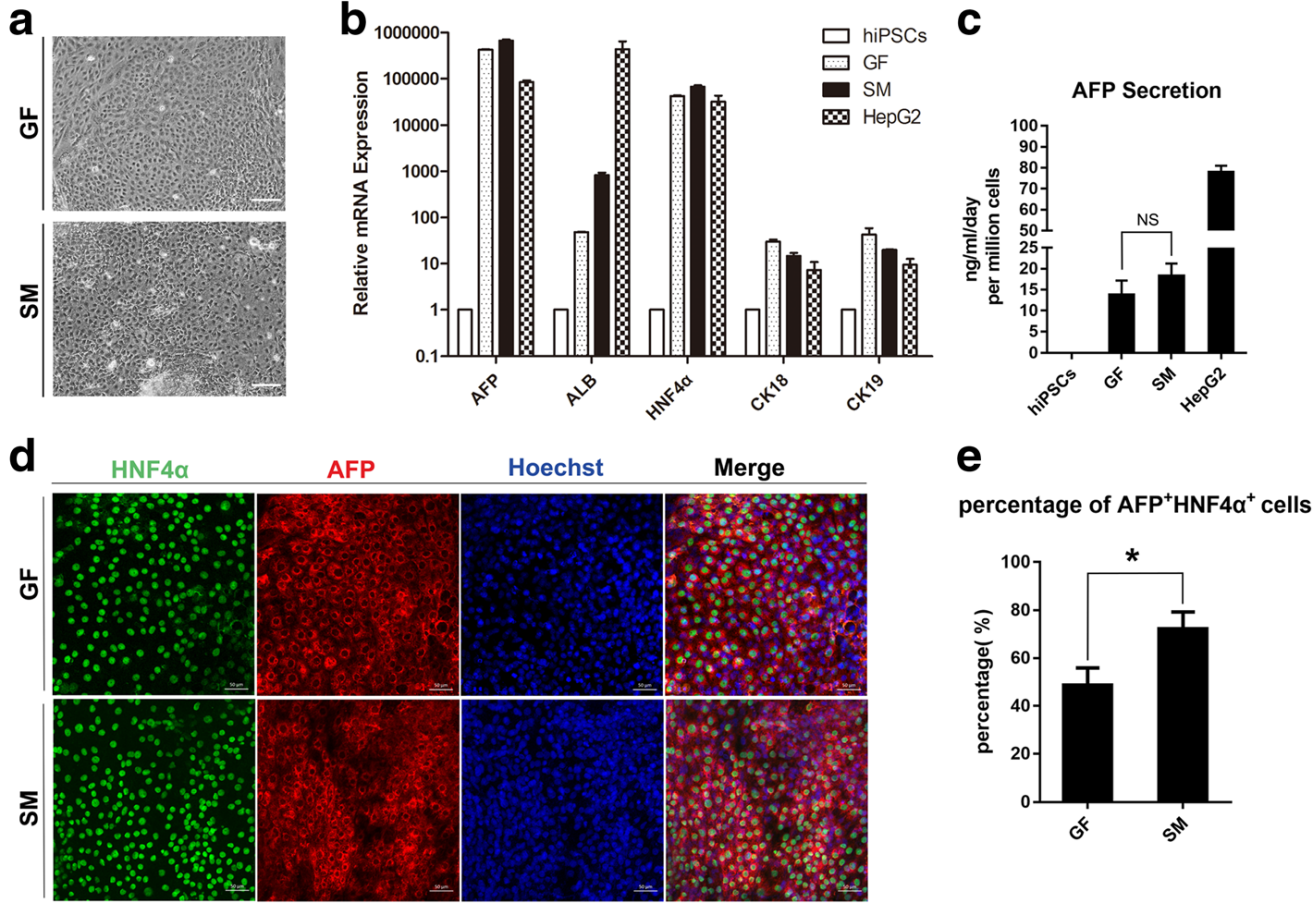
The small-molecule cocktail efficiently induces the formation of hepatic progenitors from definitive endoderm. **a** Phase contrast images showing morphology of cells in hepatic progenitor stage induced with small molecules (*lower panel*) or for 8 days with growth factors activin A, BMP-4 and FGF-4 (*upper panel*). **b** q-PCR analysis of hepatic markers (AFP, ALB, and HNF4α) and biliary markers (CK18 and CK19) RNA lysates from small-molecule cocktails or growth factors induced hepatic progenitors. Undifferentiated human iPSCs and HepG2 were used as controls. **c** AFP secretion of hepatic progenitors induced by small-molecule cocktails or growth factors. Undifferentiated human iPSCs and HepG2 were regarded as controls. **d** Immunofluorescence of HNF4α and AFP of differentiated cells induced by small-molecule cocktails or growth factors. Scale bars = 50 μm. **e** Percentage of HNF4α^+^AFP^+^ in immunofluorescence of hepatic progenitors generated using small-molecule cocktails or growth factors. (^*^p value < 0.05)

When the DE cells differentiated from hPSCs were undergoing the second stage of differentiation, a gradual change in morphology was observed. The cells transformed from petal-like clusters to cuboidal shapes typical of hepatocyte precursors (Fig. 3a). Furthermore, qRT-PCR analysis demonstrated strong expression of a repertoire of hepatic progenitor markers, including hepatocyte nuclear factor 4α (HNF4α), alpha fetoprotein (AFP), cytokeratin 18 (CK18) and cytokeratin 19 (CK19) (Fig. 3b), comparable to that of bone morphogenetic protein 4 (BMP4) and fibroblast growth factor 4 (FGF4)-based growth factor (GF)-driven differentiation.

After 5 days of treatment, secreted AFP was 15.6–21.2 ng/ml/day per million cells in the conditioned medium of SM-differentiated cells, which was a little higher than that of GF-differentiated cells (Fig. 3c). The protein level of hepatic markers AFP and HNF4α was confirmed by immunofluorescence (Fig. 3d), and the percentage of AFP and HNF4α double-positive cells of the small-molecule (SM) group was more than that of GF group (about 49.7% in GF group versus 72.25% in SM group, shown in Fig. 3e), reinforcing the conclusion that our small-molecule-based differentiation protocol, using A83–01 in combination with sodium butyrate and DMSO in the absence of any growth factors, was more efficient to induce hepatic progenitor cells from DEs than growth-factor-driven protocol.

### Efficient generation of hepatic-like cells from hepatic progenitors via a chemical compound cocktail based on the small molecules FH1 and FPH1

**Fig. 4:**
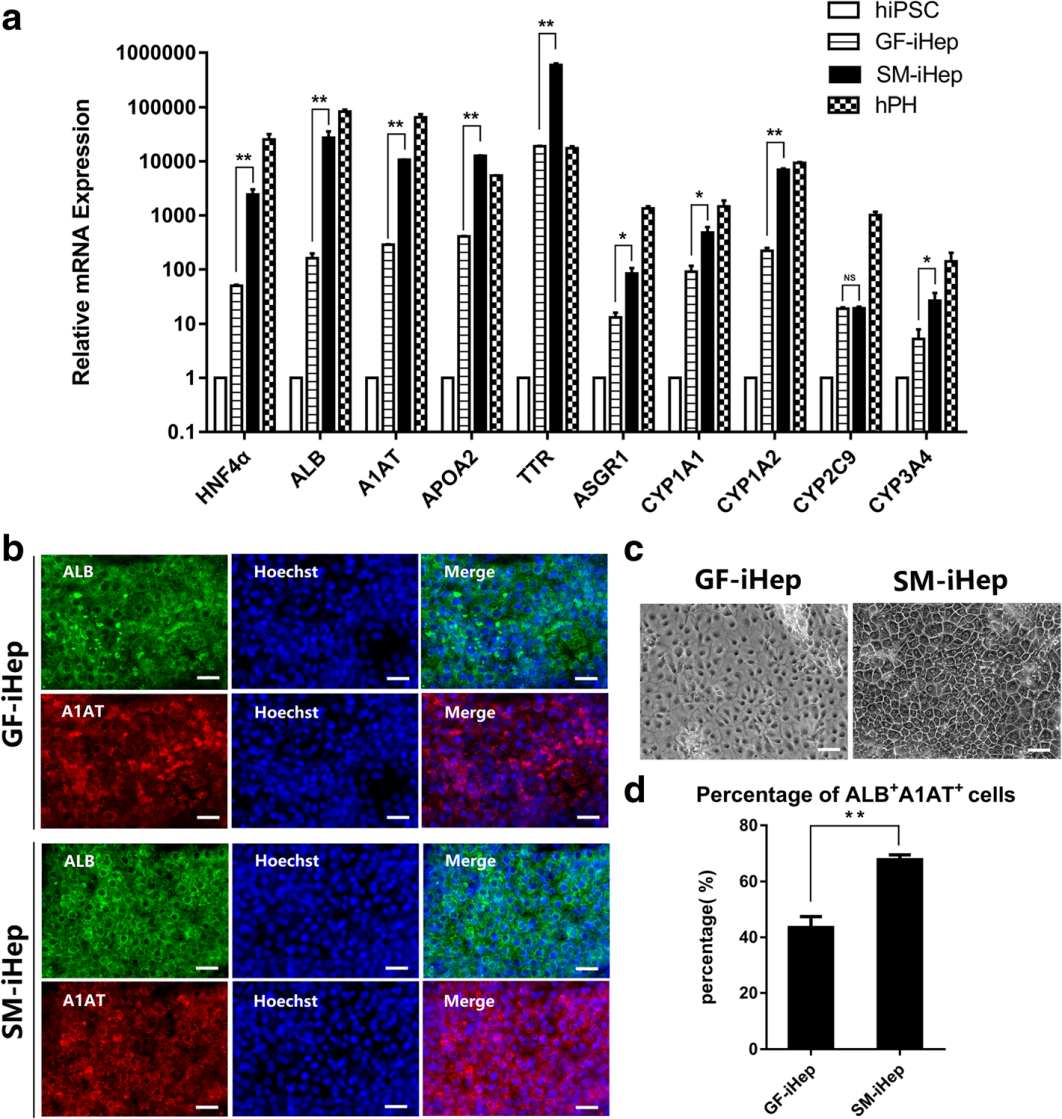
Characterization of small-molecule-induced hepatocyte-like cells. **a** qRT-PCR of hepatocyte markers at the endpoint of the small-molecule protocols with or without FH1 and FPH1. Undifferentiated human iPSCs and freshly isolated human primary hepatocytes (hPH) were used as controls. **b** Representative immunofluorescence photos of the expression of hepatocyte markers at the endpoint of the small-molecule-induced hepatocytes (SM-iHep) and growth factor-induced hepatocytes (GF-iHep). **c** Representative phase contrast photos (×200) of small-molecule-induced hepatocytes (SM-iHep) and growth factor-induced hepatocytes (GF-iHep) at day 13. Scale bars = 100 μm (**d**) Percentage of ALB^+^A1AT^+^ in immunofluorescence of hepatocyte-like cells generated using small-molecule cocktails or growth factors. (^*^p value < 0.05, ^**^p value < 0.01)

With the aim of discovering some other promising chemical compounds to replace HGF and OSM in the last stage of hepatocyte differentiation, we searched the commercial small molecule libraries and found two commercially available small molecules, named FH1 and FPH1, which were reported to be useful for human primary hepatocyte expansion in vitro [41]. These two small molecules were utilized in combination with the glucocorticoid analogs dexamethasone and hydrocortisone, which are commonly used in hepatocyte maturation. Previous publications showed that activation of the TGF-β pathway directs hepatic progenitors towards the cholangiocyte lineage and its inhibition favors the hepatocyte lineage [42–44], so TGF-β inhibitor A83–01 was continuously used to promote hepatocyte differentiation. To test whether hepatic progenitors generated in stage II could develop into hepatocytes after treatment with a small-molecule cocktail of FH1, FPH1, A83–01, dexamethasone, and hydrocortisone, the morphologic changes of the differentiated cells were monitored. Over a 5-day period, the cells became larger, angular, and cubical with bright junctions, and some microscopic fields contained multinucleated cells (Fig. 4c), showing typical hepatocyte morphology (Additional file 2: Figure S1A). At the end of the differentiation process, the gene expression of hepatocyte markers was examined by qRT-PCR. There was high expression of albumin, alpha-1 antitrypsin (A1AT), transthyretin (TTR), apolipoprotein A2 (APOA2), HNF4α as well as cytochrome P450 (CYP) enzymes CYP1A2, CYP2C9, and CYP3A4 (Fig. 4a). It is noteworthy that FH1 and FPH1 promoted hepatocyte generation (Additional file 3: Figure S2). To further analyze the properties of these small-molecule-derived hepatic-like cells, liver-specific markers were detected. The resulting induced hepatocytes exhibited the co-expression of the hepatocyte markers ALB and A1AT, which were similar with freshly isolated primary hepatocytes (Additional file 2: Figure S1B), as detected by immunofluorescence staining (Fig. 4b). Compared with the growth factor-based strategy, the small-molecule cocktail generated a higher percentage of mature hepatic-like cells (about 37.1% for growth factor group versus 67.7% for the small-molecule group), as shown in Fig. 4d.

### The small-molecule-derived hepatic-like cells possessed the function of hepatocytes

**Fig. 5:**
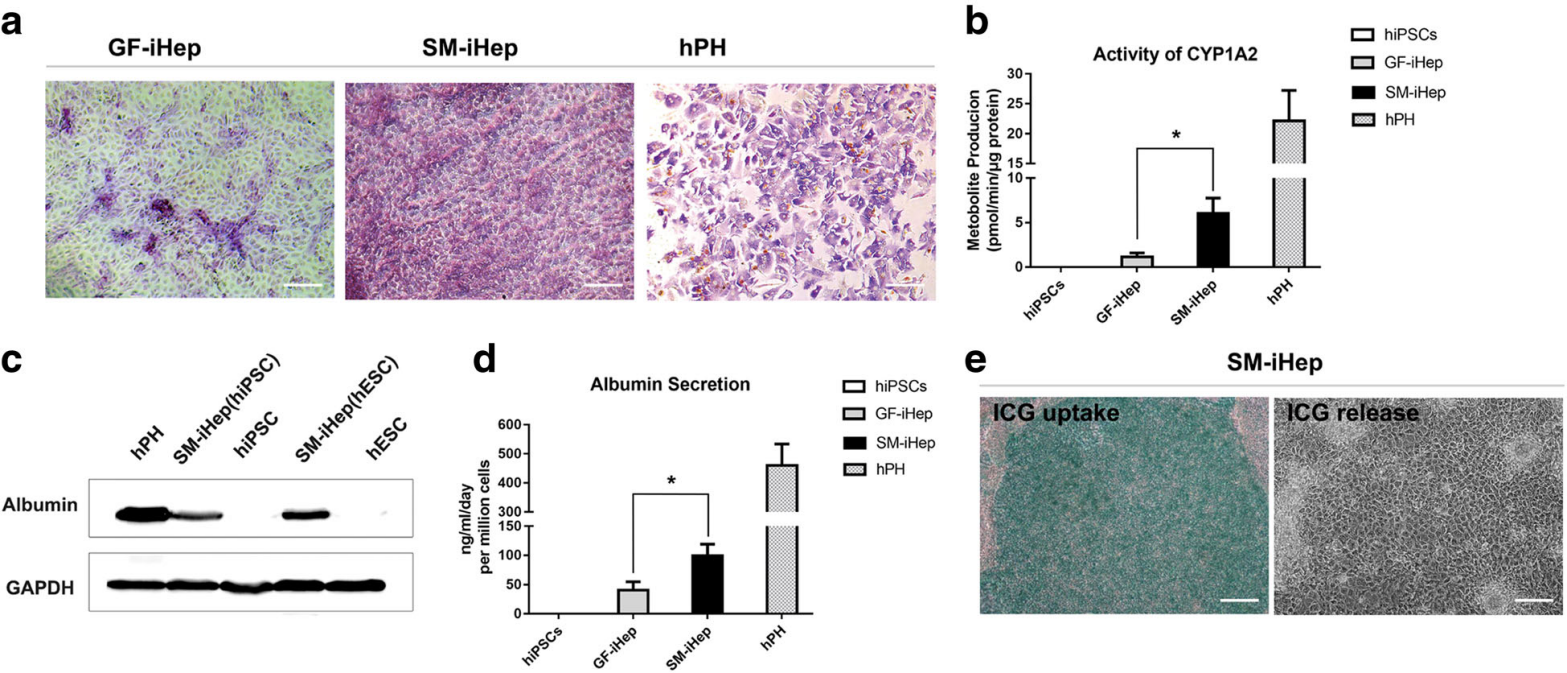
Functional analysis of small-molecule-induced hepatocyte-like cells. **a** PAS staining showing glycogen storage in small-molecule- and growth factor-induced differentiated cells. Freshly isolated human primary hepatocytes (hPH) were used as the control. Scale bars = 100 μm. **b** Cytochrome P450 1A2 activity in small-molecule- (SM-iHep) and growth factor-induced hepatocytes (GF-iHep) after induction with omeprazole (1A2). **c** Western blotting for albumin expression of small-molecule-induced hepatocyte-like cells from hESCs-H1 and hiPSCs. GAPDH was used as the loading control. **d** Albumin secretion of the differentiated cells treated with small-molecule cocktails or growth factors. Undifferentiated human iPSCs and freshly isolated human primary hepatocytes (hPH) were used as controls. **e** Analysis of ICG uptake (*left*) and ICG release 6 h later (*right*) of small-molecule-induced hepatocyte-like cells. Scale bars = 100 μm. (^*^p value < 0.05, ^**^p value < 0.01)

To examine whether these small-molecule-induced differentiated cells possess hepatocyte-specific functions, the albumin secretion were measured by western blot (Fig. 5e). Besides, compared to freshly isolated primary hepatocytes from donated liver grafts, the decrease of albumin expression revealed an obvious decline of liver function over extended culture periods in vitro [4, 5]. To quantify the albumin secretion of differentiated cells, the induced cells were cultured in six-well plates, and the supernatant was collected at day 13. The secreted albumin was significantly higher than growth factor-induced hepatocyte-like cells (about 100 ng/ml/48 h per million cells in the small-molecule group versus 40 ng/ml/48 h per million cells in the GF group) (Fig. 5d). Glycogen storage is another important characteristic of functional hepatocytes. Cells at the endpoint of our differentiation process were stained for cytoplasmic glycogen using hematoxylin. The results showed that cells were stained pink to dark red/purple, indicating their capacity to store glycogen (Fig. 5a). To test whether the hepatocytes generated using the small-molecule approach possess xenobiotic biotransformation capacity, we assessed their CYP activity. CYP1A2 is one of the essential cytochrome P450 enzymes in xenobiotic metabolism. The metabolite production by CYP1A2 in SM-iHep was more than that of GF-iHep after treatment with its specific inducer omeprazole (Fig. 5b). Uptake and release of indocyanine green (ICG) was used to characterize the function of hepatocellular uptake, conjugation, and the subsequent release of the compounds. The culture medium of induced cells was changed to differentiation medium including 1 mg/ml ICG according to the product’s manual. The differentiated cells could take up ICG and then release it 6 hours later (Fig. 5e). Hence, all these liver-specific functional tests were consistent with the expression of mature hepatocyte markers mentioned above, suggesting our proprietary small-molecule-based differentiation system was able to induce human pluripotent stem cells to generate functional hepatocytes.

### The small molecule-based differentiation approach is repeatable and universal in other tested human pluripotent stem cells

To validate the reliability and reproducibility of this differentiation protocol, we further determined whether this chemical approach could be used for other different human pluripotent stem cell lines. Therefore, the same differentiation method was applied to another two hESC lines (H1, H7) and one more human iPS cell clone reprogrammed from human fibroblasts. The results acquired from H1 and H7 were used as representative data and are in the supplementary files (Additional file 4: Figure S3, Additional file 5: Figure S4, Additional file 6: Figure S5 and Additional file 7: Figure S6).

**Fig. 6:**
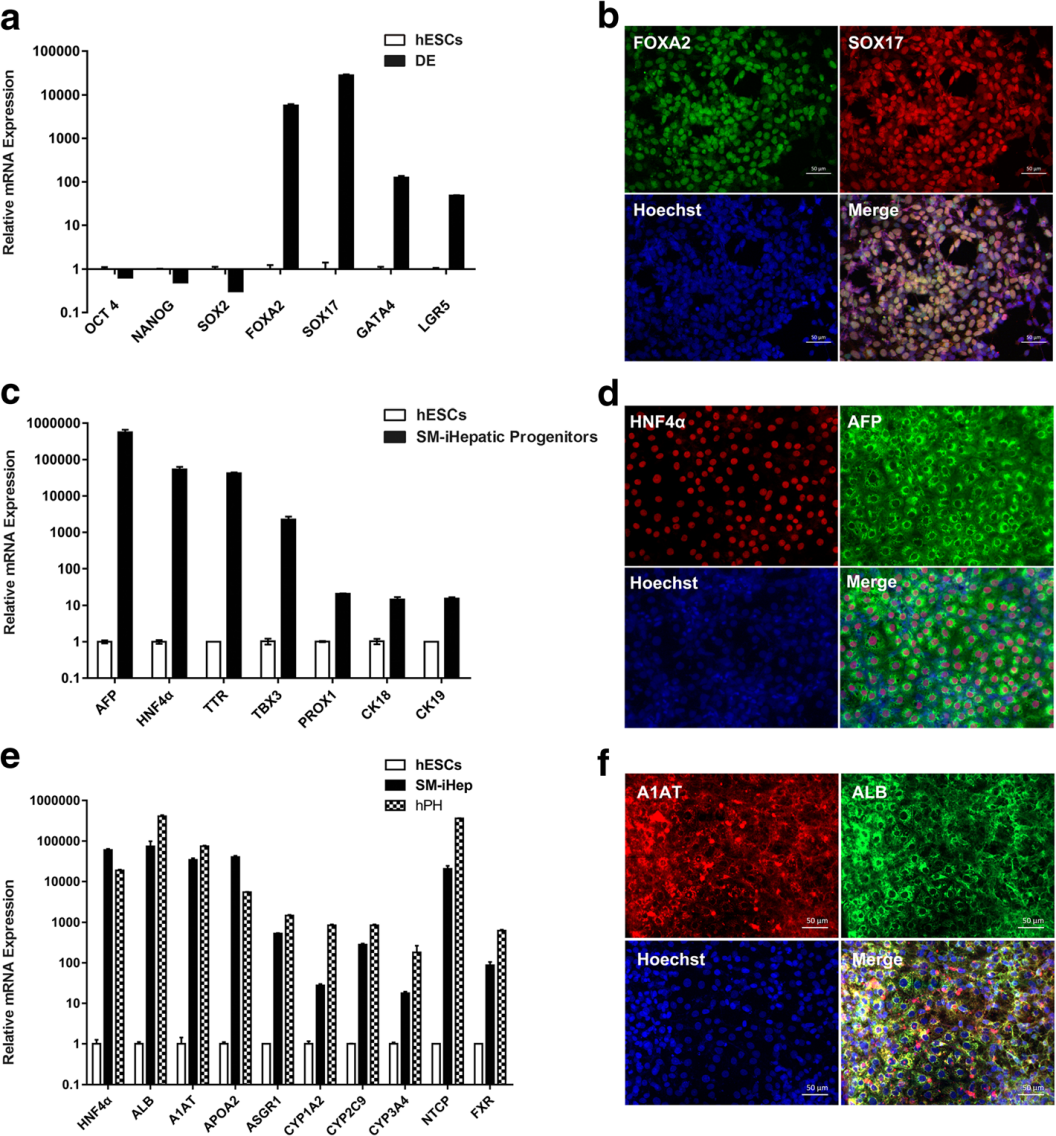
The established chemical differentiation protocol is also applicable to other human pluripotent stem cell lines. **a** qRT-PCR analysis of pluripotency and DE-specific markers in hESC-H1 differentiated cells at the endpoint of stage I. Undifferentiated hESC-H1 were used as control. **b** Immunofluorescence of FOXA2 and SOX17 in hESC-H1 differentiated cells at the endpoint of stage I. Scale bars = 50 μm. **c** Gene expression of hepatic progenitor cell markers in hESC-H1 differentiated cells at the endpoint of stage II. Undifferentiated hESC-H1 cells were regarded as control. **d** Immunofluorescence of AFP and HNF4α in hESC-H1-differentiated cells at the endpoint of stage II. Scale bars = 50 μm. **e** qRT-PCR analysis of hepatocyte markers in hESC-H1-differentiated cells at the endpoint of stage III. Undifferentiated hESC-H1 and freshly isolated human primary hepatocytes (hPH) were regarded as controls. **f** Immunofluorescence of A1AT and ALB in hESC-H1-differentiated cells at the endpoint of stage III. Scale bars = 50 μm

For initial differentiation, these additional cell lines were preconditioned with DMSO and subsequently treated with CHIR99021. Over a 72-h period, we observed a distinct morphology change from dense clones to dispersed single cells of larger size. Consistent with the alteration in the microscopy analysis, the gene expression level of a variety of definitive endoderm-specific markers was significantly upregulated, while the pluripotency transcription factors were downregulated accordingly (Fig. 6a). To confirm DE formation, we also detected the DE-specific markers FOXA2 and SOX17 at the protein level using immunofluorescence. As expected, the two DE-specific transcription factors were co-expressed in approximately 80% of the total cells (Fig. 6b). All cell lines demonstrated similar extensive changes, including the morphology and gene/protein expression levels.

Then, antagonists of TGF-β receptors and sodium butyrate were applied to H1-derived DE for hepatic specification as mentioned above. The same concentration and duration were used in the additional cell lines. At the endpoint of stage II, the hepatoblast markers AFP and HNF4α were detected by qRT-PCR and immunofluorescence (Fig. 6c and d). The acquired hepatoblasts were treated with a combination of five chemical compounds, including A83–01, dexamethasone, hydrocortisone, FPH1, and FH1. The hepatoblasts all responded well and transformed into polygonal cells, a typical morphologic appearance of hepatocytes. On day 13, the endpoint of the differentiation process, the induced cells showed quite high levels of mature hepatocyte markers, including ALB, A1AT, APOA2, CYP1A2, CYP2C9, and CYP3A4 (Fig. 6e and f). Importantly, the final differentiated cells exhibited high expression levels of Na^+^ −taurocholate co-transporting polypeptide (NTCP) and farnesoid X receptor or bile acid receptor (FXR) (Fig. 6e), which are known essential mediators for hepatitis B virus entry of hepatocytes and hence, suggesting this hepatic differentiation system may be used for liver disease modeling of viral hepatitis. Similarly, the cells could also secrete albumin, take up and release ICG, and stained positive for PAS, indicating that they obtained hepatocyte functions to some extent (similar data not shown). Taken together, all these data suggest that our chemical-based differentiation system is reliable and repeatable for multiple human pluripotent stem cell lines.

## Discussion

Orthotopic liver transplantation (OLT) remains one of the major therapeutic options for both acute and chronic liver failure. However, the patients’ need for OLT far outweighs the availability of liver grafts. Hepatocyte transplantation could be a viable alternative or adjuvant treatment to OLT. Several clinical trials and preclinical studies using animal models support the safety and preliminary efficacy of using hepatocyte transplantation therapeutically [2]. One major obstacle to the widespread clinical application of hepatocyte transplantation is the limited availability and, in some cases, marginal quality of isolated cells from donor livers. The need to expand primary hepatocytes derived from donor livers could be replaced by using human PSCs to generate hepatocytes. The generation of clinically and scientifically useful hepatocytes from human PSCs requires the availability of completely defined culture conditions that support efficient and reproducible differentiation of human PSCs into the hepatic lineage. Most of the reported procedures that have been applied to the differentiation of human ESCs and iPSCs generally include steps in which poorly defined components or expensive cytokines are added into the culture medium, and thus, potential problems exist for these cells to be used therapeutically. Therefore, we attempted to optimize the differentiation procedure and to eliminate the use of embryoid bodies, undefined culture medium components, and cytokines.

**Fig. 7:**
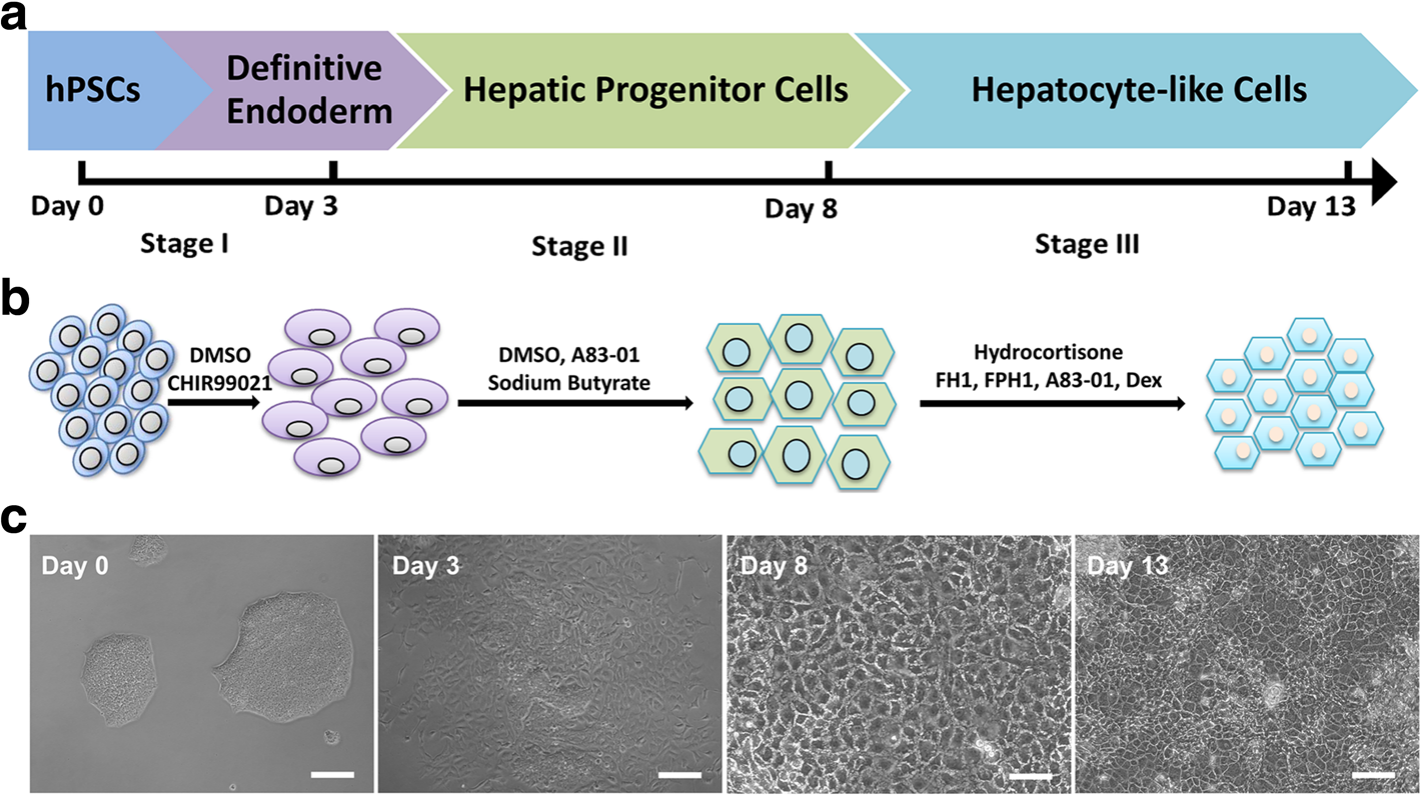
Schematic diagram of the three-stage stepwise strategy to induce hPSC differentiation into hepatocytes. **a**, **b** The detailed hepatocyte differentiation protocol we developed using pure small molecules. **c** Representative images showing sequential morphologic changes during the differentiation process. Scale bars = 100 μm

In the present study, an efficient and rapid differentiation protocol was established for generating functional hepatocyte-like cells from multiple human pluripotent stem cells by the sequential addition of small-molecule cocktails to the medium (Fig. 7). These small-molecule-induced hepatocytes showed the typical morphologic features of hepatocytes. Importantly, these induced hepatocytes expressed a panel of hepatic lineage markers. The hepatocytes also exhibited functional hepatic characteristics, such as glycogen storage, ICG uptake and release, and albumin secretion. Because our differentiation system is efficient and expedited, it may be a promising method to obtain large quantities of functional hepatocytes for future clinical applications. The advantages of our current study compared with previous reports lies in the unique chemically defined differentiation strategy without the use of any growth factors, and thus, this strategy is a defined chemical approach in the true sense. Compared with other small-molecule-based protocols [34], our approach is different in many key details. Furthermore, the process of our differentiation system is more rapid and convenient than the published protocols [26], and thus may be more suitable for clinical translation. In terms of the whole differentiation time, our procedure only takes 13 days, while other groups usually needed a minimum of 15 days or even a month. Additionally, our small-molecule-driven differentiation system could be used in both human ESCs and iPSCs. Lastly, this chemical differentiation approach is cheaper than other strategies using growth factors, which is essential for future pharmacology and clinical applications [28, 45].

All the data we acquired demonstrates that hepatocyte generation in culture plates partly follows the physiologic development stages in vivo. More importantly, understanding the fundamental mechanisms that direct liver organogenesis has laid the basis for the rational stepwise differentiation of pluripotent stem cells into mature hepatocytes. Multiple signaling pathways are involved in the complex processes of liver development, including the Wnt/β-catenin pathway, the FGF/MAPK pathways, the TGF-β pathway, and the hepatocyte growth factor (HGF)/c-Met pathway [43, 44]. On the basis of these recognized signaling pathways, we carefully selected a number of promising small molecules and examined their effects by performing numerous experiments.

The Wnt/β-catenin signaling pathway regulates SOX17 expression during DE formation [32, 38]. Based on this rationale, Hay et al. reported that Wnt pathway activation through the cytokine wnt3a is required for DE formation from human ESCs [31]. We hypothesized that Wnt pathway activation through CHIR99021, a GSK3β inhibitor, could substitute that of wnt3a to initiate DE formation. A number of other publications have also reported that the inhibition of GSK3β could be useful for DE formation [33, 34].

DE cells from human PSCs could be acquired using a quite simple and efficient protocol, which would ensure subsequent hepatic specification and hepatocyte maturation when used in combination with other small molecules. In the past few years, scholars have found that DMSO and sodium butyrate could direct DE differentiation toward the hepatic lineage [22, 40]. Evidence shows that both DMSO and sodium butyrate can play a part in epigenetic modifications and generally regulate gene expression, indicating that they are nonspecific agents. Li et al*.* revealed that sodium butyrate decreases Bmi-1, cyclin B1, and Cdk4 expression, which may be associated with hepatic differentiation [46]. However, DE differentiation can typically yield a range of developmental outcomes, including liver cells, pancreatic cells, and enterocytes, hence the need to eliminate the undesired lineages and promote the DE to differentiate exclusively toward hepatic cells. To combat this challenge, we focused on the TGF-β signaling pathway, which is essential for lineage specification. According to reported findings both in vivo and in vitro, TGF-β and BMP signaling dually specify the pancreas versus liver lineage, and each bilaterally cross-represses the alternate fate [42]. Thus, efficient liver induction requires TGF-β inhibition. Hence, we rationally designed the stage II differentiation scheme with A83–01, an inhibitor of TGF-β receptors, which was combined with DMSO and sodium butyrate as mentioned above. The three small molecules could efficiently promote hepatic specification from DE and generate hepatoblasts in 5 days.

Immediately following the generation of hepatic progenitors, it is imperative to generate mature hepatocytes. HGF and OSM are vital for the maturation of hepatocytes and there has been no commercial substitute for the two cytokines in the past years. There were two hopeful commercial small molecules, named FH1 and FPH1, which were able to induce proliferation and enhance the functions of primary human hepatocytes cultured in vitro [41]. These two chemical compounds and two other commonly used small molecules from the glucocorticoid family, dexamethasone, and hydrocortisone, were applied in the final stage of hepatocyte generation from hepatic progenitors. After 13 days of treatment, mature hepatocyte-like cells were obtained which not only expressed a variety of representative markers but also exhibited several typical functional attributes. However, further study is required to obtain perfectly functional hepatocytes for clinical use. For example, the signaling at each differentiation stage needs further delineation for a more efficient and expedited differentiation system.

As with the improvement of differentiation strategies, hepatocyte-like cells generated by most published protocols showed encouraging results of functions in vitro, and even the ability to repopulate in the liver and execute functions after being transplanted into animal models [10, 15, 47]. Nevertheless, the maturation of most cells differentiated so far are still needed to compare to their in vivo counterparts. Small molecules are showing great potential to modulate cell fates and improve their functions. By recapitulating organ development in vivo, sequential addition of specific small molecules to PSCs has yielded a number of target cell types, such as neurons, cardiomyocytes, retinal pigment epithelium, and so on [28]. In addition to stem cell differentiation, small molecules have also been successfully utilized in reprogramming, trans-differentiation and maintaining pluripotency [27, 29]. Most recently, scientists found that small molecules could induce cell fate conversion between somatic cells, a direct process that bypasses the pluripotent state and could be even faster in generating the desired cell types [30, 48]. For example, fibroblasts were converted to hepatocyte-like cells with the treatment of small molecules combined with growth factors [30]. It is speculated that more molecules are waiting to be discovered and completely replace growth factors. While considering applications of these small-molecule-derived cells in the future, significant efforts should be invested into improvement of their functions and precise control of their fates. For these ultimate purposes, identification of more novel molecules with specific targets by high-throughput screening will continue to serve as a powerful strategy. In addition, more and more biomaterials are playing essential roles in the field of stem cell research since they can exert biochemical effects and provide physical structure support. In conclusion, on the basis of a deeper understanding of stem cell biology, more efforts are needed to optimize current chemical approaches for better manipulation of stem cell fate.

## Conclusions

In summary, we have established a three-step strategy to induce the direct differentiation of various human PSCs toward functional hepatocyte-like cells using pure small-molecule-composed cocktails. This differentiation procedure is an efficient, reproducible, stable, economical, and timesaving method to generate scalable hepatocyte-like cells. More efforts are underway to produce an ideal hepatic induction strategy for future individualized hepatocyte transplantation and pharmaceutical screening.

## Additional files

Additional file 1: **Table S1.** Small molecules used for hepatic differentiation. **Table S2.** Primers used to amplify the transcripts during real-time quantitative PCR. **Table S3.** Antibodies used for detection. (DOCX 21 kb)

Additional file 2: **Figure S1.** (A) Representative phase contrast images showing the morphology of freshly isolated human primary hepatocytes. (B) Immunofluorescence images of ALB and A1AT in human primary hepatocytes. Scale bars = 100 μm. (TIFF 325 kb)

Additional file 3: **Figure S2.** qRT-PCR of hepatocyte markers at the endpoint of the small-molecule protocols with or without FH1 and FPH1. Undifferentiated human iPSCs was regarded as control. SM1 represents protocol composed of A83–01, dexamethasone and hydrocortisone. (^*^p value < 0.05, ^**^p value < 0.01). (TIFF 328 kb)

Additional file 4: **Figure S3.** qRT-PCR for pluripotency genes using RNA lysates from hESC-H7 at the endpoint of stage I. Undifferentiated hESC-H7 was used as control. (TIFF 57 kb)

Additional file 5: **Figure S4.** qRT-PCR for DE specific makers using RNA lysates from hESC-H7 at the endpoint of stage I. Undifferentiated hESC-H7 was used as control. (TIFF 79 kb)

Additional file 6: **Figure S5.** qRT-PCR for hepatic makers using RNA lysates from hESC-H7 at the endpoint of stage I and II. Undifferentiated hESC-H7 was used as control. (TIFF 376 kb)

Additional file 7: **Figure S6.** qRT-PCR for DE hepatocyte makers using RNA lysates from hESC-H7 at the endpoint of stage II and III. Undifferentiated hESC-H7 and freshly isolated human primary hepatocytes (hPH) were used as controls. (TIFF 262 kb)

## Abbreviations

A1AT: Alpha-1 antitrypsin
AFP: Alpha-fetoprotein
ALB: Albumin
APOA2: Apolipoprotein A2
BMP4: Bone morphogenetic protein 4
CK18/19: Cytokeratin 18/19
DE: Definitive endoderm
DMSO: Dimethyl sulfoxide
EB: Embryoid body
ELISA: Enzyme-linked immunosorbent assay
FBS: Fetal bovine serum
FGF4: Fibroblast growth factor 4
FXR: Farnesoid X receptor or bile acid receptor
GSK-3β: Glucogen synthase kinase 3β
hESC: Human embryonic stem cells
HGF: Hepatocyte growth factor
HLC: Hepatocyte-like cell
HNF4α: Hepatocyte nuclear factor-4α
ICG: Indocyanine green
iPSC: Induced pluripotent stem cell
NTCP: Na + −taurocholate co-transporting polypeptide
OLT: Orthotopic liver transplantation
OSM: Oncostatin M
PAS: Periodic acid-Schiff
PSC: Pluripotent stem cell
qPCR: Quantitative PCR
SB: Sodium butyrate
SOX17: Sex determining region Y (SRY)-box 17
TGF-β: Transforming growth factor-β
TTR: Transthyretin
