# Supplementary material for: Highly efficient and expedited hepatic differentiation from human pluripotent stem cells by pure small-molecule cocktails
Source: Stem Cell Res Ther. 2018 Mar 9;9:58. doi: 10.1186/s13287-018-0794-4 (PMC5845228; doi:10.1186/s13287-018-0794-4)
Supplement: Supplementary file 1 — Table S1. Small molecules used for hepatic differentiation. Table S2. Primers used to amplify the transcripts during real-time quantitative PCR. Table S3. Antibodies used for detection. (DOCX 21 kb) [file 13287_2018_794_MOESM1_ESM.docx]

**Additional file 1**

**Table S1. Small molecules used for hepatic differentiation**

| Name |  | Distributor (Catalog Number) |
| --- | --- | --- |
| CHIR99021  DMSO  Sodium Butyrate  A83-01  FH1(BRD-K4477)  FPH1 (BRD-6125)  Dexamethasone  Hydrocortisone |  | Selleck(S1263)  Sigma Aldrich (2650)  Sigma Aldrich (B5887)  STEMGENT( 04-0014)  Selleck(S7450)  Selleck(S7451)  Selleck(S1322)  Selleck(S1696) |

| **Table S2. Primers used to amplify the transcripts during real-time quantitative PCR.** | | |
| --- | --- | --- |
| Gene | Sequence (5′ to 3′) | |
| GAPDH | | Forward: ACCATCTTCCAGGAGCGAGAT  Reverse: ATGACGAACATGGGGGCATC |
| OCT4 | Forward: CAGTGCCCGAAACCCACAC  Reverse: GGAGACCCAGCAGCCTCAAA | |
| NANOG | Forward: TTTGTGGGCCTGAAGAAAACT  Reverse: AGGGCTGTCCTGAATAAGCAG | |
| SOX2  FOXA2 | Forward: CCATGCAGGTTGACACCGTTG  Reverse: TCGGCAGACTGATTCAAATAATACAG  Forward: TGCACTCGGCTTCCAGTATG  Reverse: CGTGTTCATGCCGTTCATCC | |
| GATA4 | Forward: TCCAAACCAGAAAACGGAAGC  Reverse: GCCCGTAGTGAGATGACAGG | |
| CXCR4 | Forward: TCCATTCCTTTGCCTCTTTTGC  Reverse: TGTCCGTCATGCTTCTCAGTT | |
| SOX17  GSC  BRA | Forward: GGCGCAGCAGAATCCAGA  Reverse: CCACGACTTGCCCAGCAT  Forward: AGGAGAAAGTGGAGGTCTGGT  Reverse: CTGTCCGAGTCCAAATCGCT  Forward: GGGTACTCCCAATCCTATTCTGAC  Reverse: ACTGACTGGAGCTGGTAGGT | |
| HAND1  BMP5  AFP  HNF4α  CK18  CK19  TTR  TBX3  ALB  A1AT  APOA2  CYP2B6  CYP1A2  ASGR1  CYP2C9  CYP3A4  GAP43  NTCP  FXR  ZIC1  LGR5 | Forward: AGGCTGAACTCAAGAAGGCG  Reverse: AGCCGGTGCGTCCTTTAATC  Forward: CGTGAGAGCAGCCAACAAAC  Reverse: TGGTGCTATAATCCAGTCCTGC  Forward: AAATGCGTTTCTCGTTGCTT  Reverse: GCCACAGGCCAATAGTTTGT  Forward: ACTACATCAACGACCGCCAGT  Reverse: ATCTGCTCGATCATCTGCCAG  Forward: CACAGTCTGCTGAGGTTGGA  Reverse: GAGCTGCTCCATCTGTAGGG  Forward: TGAGGAGGAAATCAGTACGCT  Reverse: CGACCTCCCGGTTCAATTCT  Forward: CATGGGCTCACAACTGAGGA  Reverse: TTGGCTGTGAATACCACCTCTG  Forward: CTGCAGTCCATGAGGGTGTT  Reverse: ATGCTCCTCTTTGCTCTCGG  Forward: GCACAGAATCCTTGGTGAACAG  Reverse: ATGGAAGGTGAATGTTTCAGCA  Forward: AGGTGCCTATGATGAAGCGT  Reverse: TGGCAGACCTTCTGTCTTCATT  Forward: GTTCGGAGACAGGCAAAGGA  Reverse: TCAAAGTAAGACTTGGCCTCGG  Forward: TCTCCTTAGGGAAGCGGATTTG  Reverse: GCAGGAAGCGGATCTGGTAT  Forward: ATGTGAGCAAGGAGGCTAAGG  Reverse: GGCAGTCTCCACGAACTCA  Forward: CACCATCAGCTCAGAAAAGGGC  Reverse: TTCTTCCCACATTGCCTCCCTG  Forward: CAAGATTTTGAGCAGCCCCTG  Reverse: TGGTTGTGCTTTTCCTTCTCCA  Forward: GTGGGGCCTTTGTCAGAACT  Reverse: TGGGCAAAGTCACAGTGGAT  Forward: TTGACTTTCTGGATTTCAAGGGTTG  Reverse: CCACGGAAGCTAGCCTGAAT  Forward: CCTCAAATCCAAACGGCCAC  Reverse: TGGCAGAGAGAACTGTGACG  Forward: ATGCAAAGAGATGGGAATGTTGG  Reverse: TCGCAAGTCACGACCTTCAC  Forward: ACATGAAGGTCCACGAATCCTC  Reverse: AGGGCGATAAGGAGCTTGTG  Forward: GAGGAGTTACGTCTTGCGGG  Reverse: AAACAGCTTGGGGGCACATA | |

**Table S3. Antibodies used for detection.**

| Marker (Species) | Application (Dilution) | Distributor (Catalog Number) |
| --- | --- | --- |
| Primary antibodies: |  |  |
| Nanog(mouse)  Oct3/4 (mouse)  FoxA2/HNF3β (rabbit) | IF (1:500)  IF (1:500)  IF (1:400) | Santa Cruz ( sc-293121)  Santa Cruz ( sc-5279)  CST( #8186) |
| Sox17 (rabbit)  PE Mouse anti-Human FoxA2  PerCP-Cy™5.5 Mouse anti-Human Sox17  APC Mouse Anti-Human CD184/CXCR4 | IF (1:500)  FCM (5μl/test)  FCM (5μl/test)  FCM (5μl/test) | CST ( #81778)  BD Pharmingen (561589)  BD Pharmingen (562387)  BD ( 560936 ) |
| Anti-human/mouse α-Fetoprotein Antibody  Human alpha 1-Antitrypsin Antibody (Mouse)  Anti-human/mouse Albumin Antibody  E-Cadherin (24E10) Rabbit mAb  HNF4α Rabbit mAb  Human Albumin Antibody(Goat)  GAPDH (14C10) rabbit Ab | IF (1:100)  IF (1:100)  IF (1:100)  IF (1:1000)  IF (1:1000)  WB (1:1000)  WB (1:1000) | R&D (MAB1368 )  R&D ( MAB1268 )  R&D ( MAB1455 )  CST (#3195)  CST (#3113)  BETHYL ( A80-129A )  CST (sc-659) |
| Secondary antibodies:  mouse anti-rabbit IgG-HRP Antibody  mouse anti-goat IgG-HRP Antibody  Donkey anti-Rabbit IgG Secondary Antibody  Donkey anti-Rabbit IgG Secondary Antibody  Donkey anti-Mouse IgG Secondary Antibody  Donkey anti-Mouse IgG Secondary Antibody  Donkey anti-Goat IgG Secondary Antibody  Donkey anti-Goat IgG Secondary Antibody | WB(1:5000)  WB(1:5000)  IF (1:1000)  IF (1:1000)  IF (1:1000)  IF (1:1000)  IF (1:1000)  IF (1:1000) | Santa Cruz (sc-2357)  Santa Cruz (sc-2354)  ThermoFisher Scientific(A-21206)  ThermoFisher Scientific(A-21207)  ThermoFisher Scientific(A-21202)  ThermoFisher Scientific(A10036)  ThermoFisher Scientific(A-11058)  ThermoFisher Scientific(A-21432) |
