# Supplementary figures and images for: Highly efficient and expedited hepatic differentiation from human pluripotent stem cells by pure small-molecule cocktails
Source: Stem Cell Res Ther. 2018 Mar 9;9:58. doi: 10.1186/s13287-018-0794-4 (PMC5845228; doi:10.1186/s13287-018-0794-4)

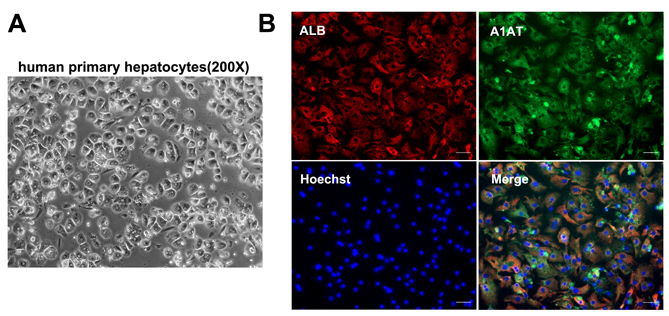

Supplement: Supplementary file 2 — Figure S1. (A) Representative phase contrast images showing the morphology of freshly isolated human primary hepatocytes. (B) Immunofluorescence images of ALB and A1AT in human primary hepatocytes. Scale bars = 100 μm. (TIFF 325 kb) [file 13287_2018_794_MOESM2_ESM.tif]

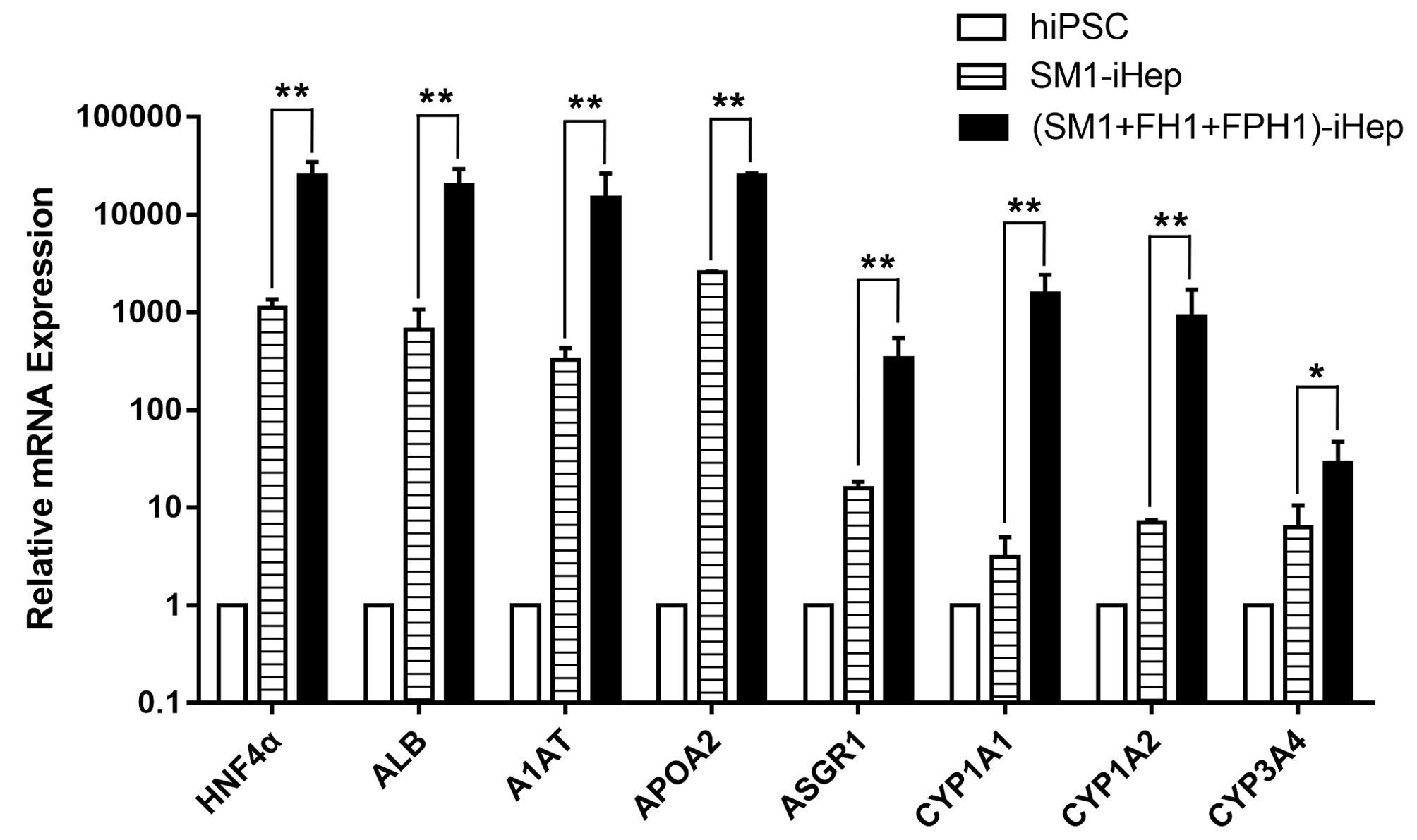

Supplement: Supplementary file 3 — Figure S2. qRT-PCR of hepatocyte markers at the endpoint of the small-molecule protocols with or without FH1 and FPH1. Undifferentiated human iPSCs was regarded as control. SM1 represents protocol composed of A83–01, dexamethasone and hydrocortisone. (*p value < 0.05, **p value < 0.01). (TIFF 328 kb) [file 13287_2018_794_MOESM3_ESM.tif]

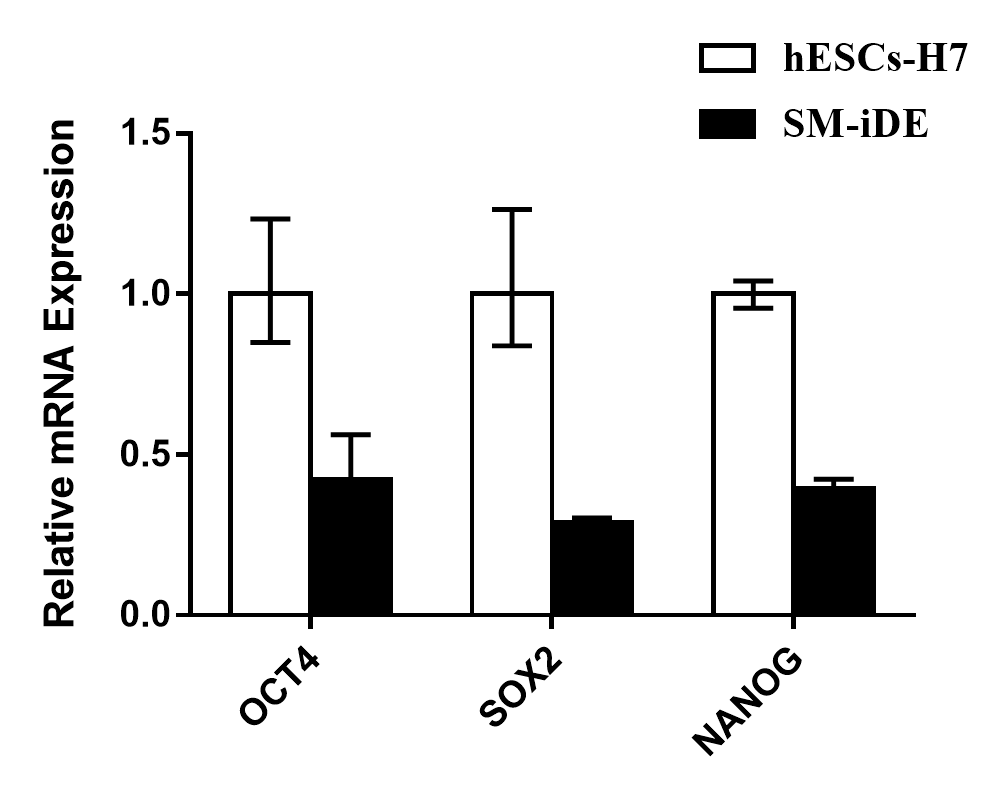

Supplement: Supplementary file 4 — Figure S3. qRT-PCR for pluripotency genes using RNA lysates from hESC-H7 at the endpoint of stage I. Undifferentiated hESC-H7 was used as control. (TIFF 57 kb) [file 13287_2018_794_MOESM4_ESM.tif]

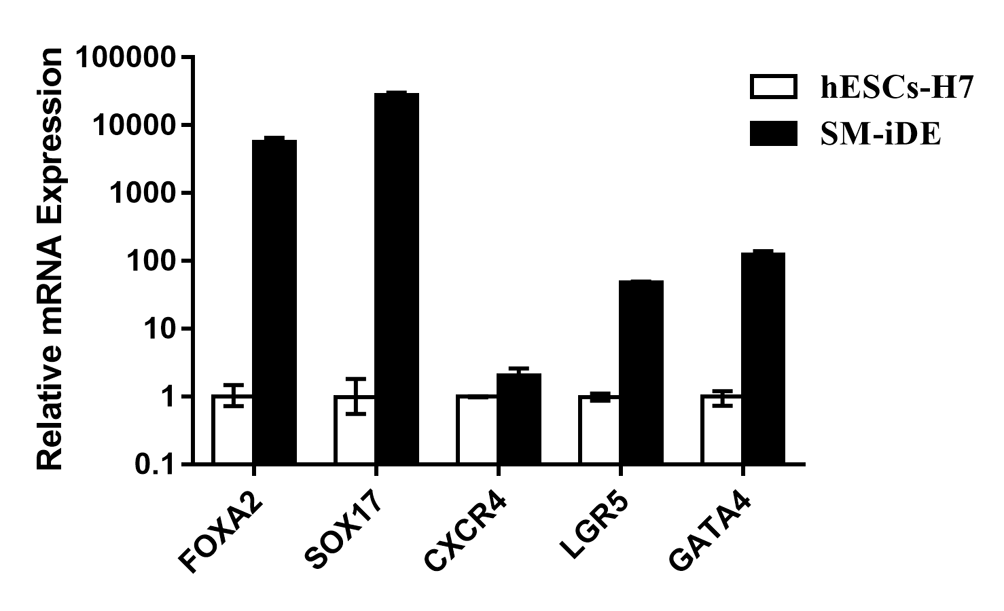

Supplement: Supplementary file 5 — Figure S4. qRT-PCR for DE specific makers using RNA lysates from hESC-H7 at the endpoint of stage I. Undifferentiated hESC-H7 was used as control. (TIFF 79 kb) [file 13287_2018_794_MOESM5_ESM.tif]

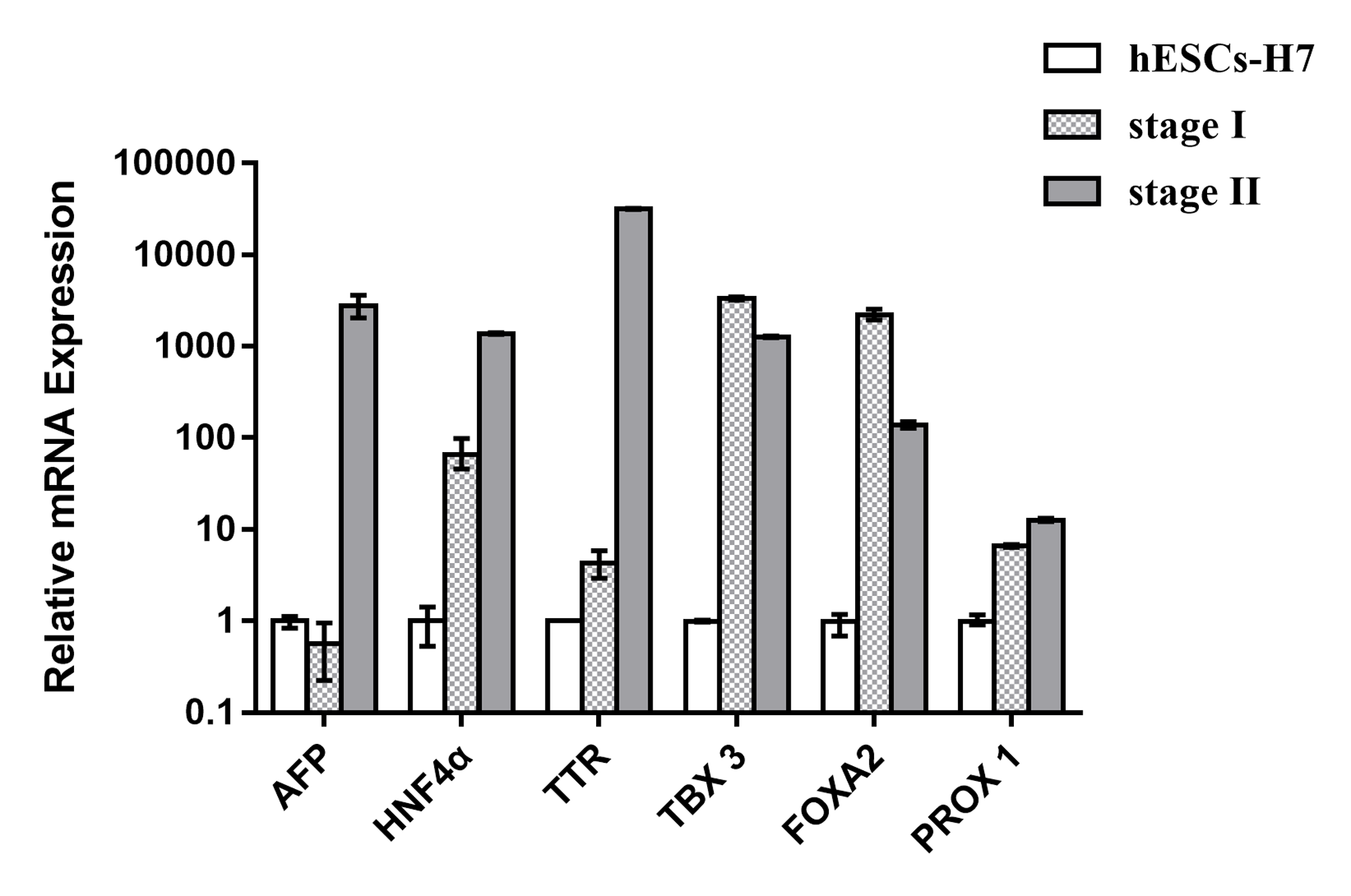

Supplement: Supplementary file 6 — Figure S5. qRT-PCR for hepatic makers using RNA lysates from hESC-H7 at the endpoint of stage I and II. Undifferentiated hESC-H7 was used as control. (TIFF 376 kb) [file 13287_2018_794_MOESM6_ESM.tif]

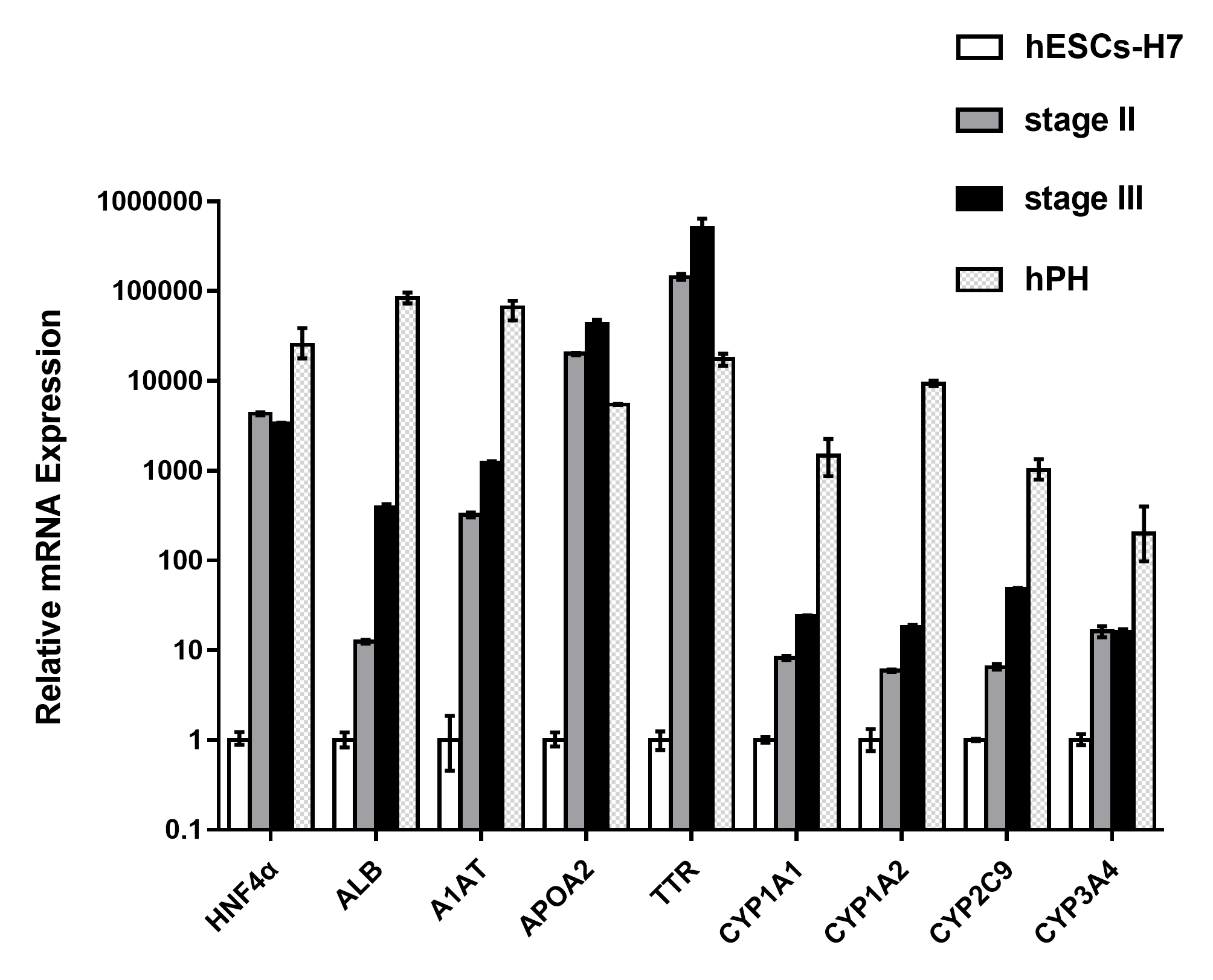

Supplement: Supplementary file 7 — Figure S6. qRT-PCR for DE hepatocyte makers using RNA lysates from hESC-H7 at the endpoint of stage II and III. Undifferentiated hESC-H7 and freshly isolated human primary hepatocytes (hPH) were used as controls. (TIFF 262 kb) [file 13287_2018_794_MOESM7_ESM.tif]
